# Human Demonstration Does Not Facilitate the Performance of Horses (*Equus caballus*) in a Spatial Problem-Solving Task

**DOI:** 10.3390/ani8060096

**Published:** 2018-06-13

**Authors:** Joan-Bryce Burla, Janina Siegwart, Christian Nawroth

**Affiliations:** 1 Centre for Proper Housing of Ruminants and Pigs, Federal Food Safety and Veterinary Office FSVO, Agroscope Tänikon, 8356 Ettenhausen, Switzerland; 2 Kantonsschlue Kollegium Schwyz, Kollegiumstrasse 24, 6430 Schwyz, Switzerland; janina.siegwart@bluewin.ch; 3 Leibniz Institute for Farm Animal Biology, Institute of Behavioural Physiology, Wilhelm-Stahl-Allee 2, 18196 Dummerstorf, Germany

**Keywords:** detour task, equids, social cognition, social learning, spatial cognition

## Abstract

**Simple Summary:**

Horses were confronted with a spatial problem-solving task in which they had to detour an obstacle. Individuals that observed a human demonstrating how to solve the task did not solve the task more often or faster compared with a control group without demonstration. However, horses of both the treatment and control group detoured the obstacle faster over trials. Together with previous research, our results illustrate that horses do not seem to rely on social information when solving a spatial problem-solving task.

**Abstract:**

Horses’ ability to adapt to new environments and to acquire new information plays an important role in handling and training. Social learning in particular would be very adaptive for horses as it enables them to flexibly adjust to new environments. In the context of horse handling, social learning from humans has been rarely investigated but could help to facilitate management practices. We assessed the impact of human demonstration on the spatial problem-solving abilities of horses during a detour task. In this task, a bucket with a food reward was placed behind a double-detour barrier and 16 horses were allocated to two test groups of 8 horses each. One group received a human demonstration of how to solve the spatial task while the other group received no demonstration. We found that horses did not solve the detour task more often or faster with human demonstration. However, both test groups improved rapidly over trials. Our results suggest that horses prefer to use individual rather than social information when solving a spatial problem-solving task.

## 1. Introduction

The management of horses is key to providing them with adequate welfare [1,2]. An important role in these management practices, such as handling and training, is the horses’ ability to adapt to new environments and to acquire new information, either individually or from others [3,4]. In the context of horse handling, social learning from humans could help to facilitate management practices but research on this topic is limited (but see [5]). As horses often experience frequent interactions with humans, either due to training or general husbandry practices, potential heterospecific information transfer from handlers to horses might thus help to improve their welfare [6].

Animals are able to obtain solutions to novel problems by trial-and-error learning or via social learning, i.e., by observing or interacting with other individuals [7,8]. However, research on social learning in horses found contradictory results on their ability to solve novel problems by the observation of conspecific demonstrators. Horses that observed a conspecific manipulating a test apparatus to receive a food reward spent more time close to the test apparatus but did not learn to operate it more quickly compared with horses that did not receive a demonstration [9]. In addition, horses that observed a demonstrator horse solving a spatial task were not faster in solving this task than horses that did not receive a social demonstration [10,11]. Age and dominance rank of the demonstrator and observer can affect the transmission of social information. For instance, younger, lower-ranking, and more explorative horses showed an improved performance in an instrumental manipulation task when observing a conspecific solving this task [12]. Horses also copied specific following behaviours towards humans when a familiar and dominant conspecific demonstrator followed the path of a human handler, but not when the demonstrator was a subordinate or unknown conspecific [13]. However, older and dominant demonstrators did not enhance the performance of observer horses in a spatial problem-solving task in comparison to observer horses with age-matched demonstrators or control horses without a demonstration [10]. In conclusion, horses’ performance in instrumental but not spatial tasks is affected by a previous demonstration of a conspecific. Given these ambiguous results, researchers have stressed that tasks must be ecologically relevant and, further, that dominance and age effects should be taken into account in social learning [14].

Social learning is not restricted to conspecifics but can also take place with heterospecifics (e.g., [15]). Domestic animals, especially dogs, have a high inclination to interact and communicate with humans [16,17,18]. This adaptation might also facilitate their ability to learn from humans through observation. When horses were given the opportunity to frequently observe a human solving an instrumental task, more individuals learned the task and also learned it faster than horses that did not receive a human demonstration [5]. However, there is currently no research on the impact of human demonstration on horses in spatial tasks.

Spatial problem-solving tasks are often used to investigate social learning from conspecifics and heterospecifics [19,20,21]. For example, the ability of dogs to solve tasks in which they have to walk around obstacles to reach a food reward has been widely investigated in the context of social learning [19]. Dogs detoured a V-shaped barrier faster after they observed a human demonstrator carrying a food container before solving the task themselves compared to receiving no demonstration [19]. However, it appears that, at least for dogs, stimulus enhancement effects could explain their improved performance; dogs appear to simply follow the movement of the food container even when no human is present [22]. Although horses can solve these detour tasks on an individual level [23,24,25], a first study on the use of social information in this specific task indicates that horses do not benefit from a demonstration by a conspecific [10]. However, whether observing a demonstration by a human affects the performance of horses in this task has not been investigated yet.

In the present study, we investigated the effect of a human demonstrator on the performance of horses in a spatial problem-solving task. We presented horses with a series of 10 trials with either the presence or absence of a human demonstrator. We expected horses which observed a human demonstration to perform better in the detour task than horses that did not observe a demonstration [19,20]. We further expected horses to improve over trials [19,23,25], independently of the presence or absence of a human demonstrator.

## 2. Materials and Methods

### 2.1. Subjects and Housing

The study was conducted with 16 horses at a riding stable in Switzerland during August and September 2012. The 9 mares and 7 geldings were between 4 and 19 years ($\bar{x}$ ± SD: 9.9 ± 4.9) old and of various common riding horse breeds. All horses were owned by private owners and used to being handled and exercised on a daily basis. They were housed in individual box stalls (3.5 × 3.5 m) with straw bedding, had access to a paddock or pasture several times per week, and feeding of hay and concentrate took place 2 and 3 times a day, respectively. Routine care remained unchanged during the period of experiments and was provided by stable employees and their owners.

### 2.2. Ethical Note

Animal care and experimental procedures were in accordance with the guidelines for the treatment of animals in behavioural research and teaching by the Association for the Study of Animal Behaviour [26] and the Swiss animal welfare legislation [27,28]. Daily experimental procedures took place in a familiar environment and lasted no more than 20 min per horse. The experiments would have been terminated if a horse had shown signs of stress (e.g., increased alertness, locomotion, or vocalization) but all individuals adapted well and participated voluntarily.

### 2.3. Experimental Setup

**Figure 1:**
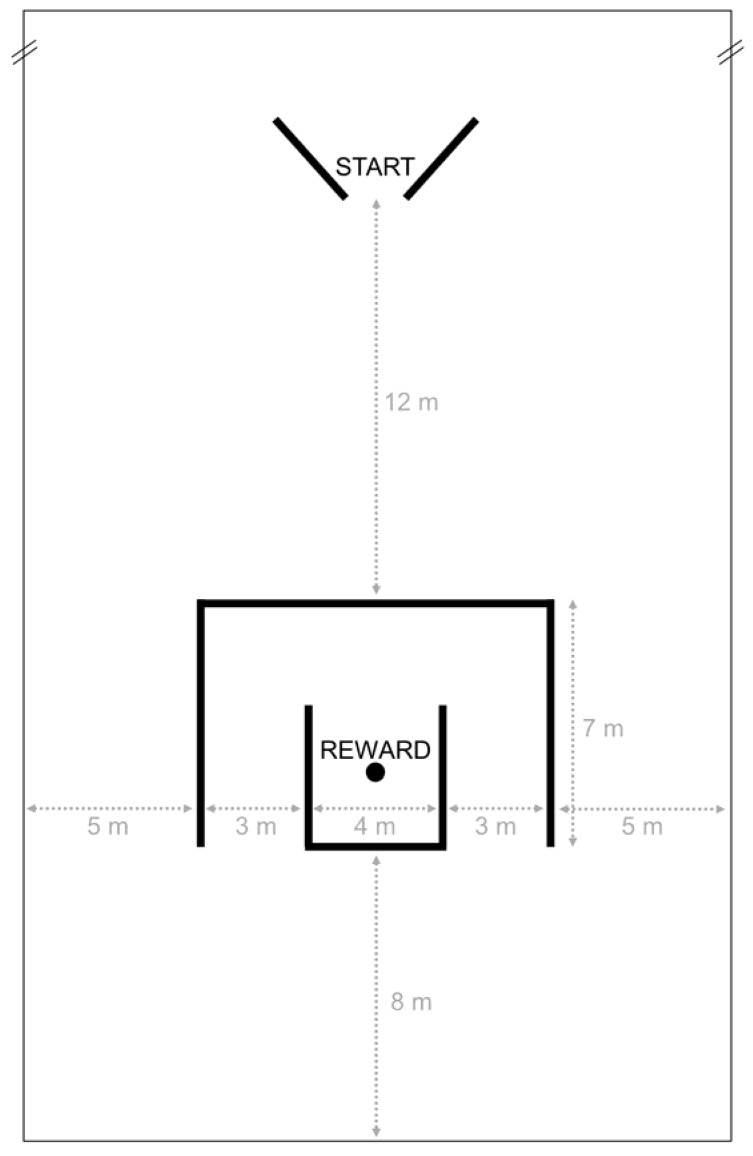
Overview of the experimental setup in the test arena.

**Figure 2:**
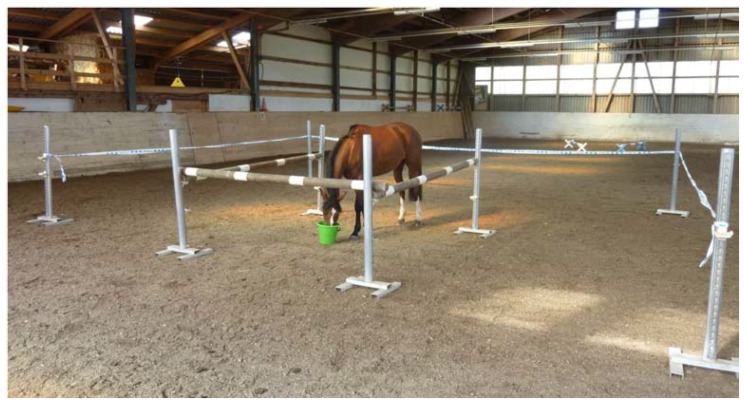
Horse feeding from the rewarded bucket after successfully completing the detour task.

The experiments were conducted at the stable’s indoor riding arena (20 × 40 m), which was familiar to all horses. A double-detour task was set up by two nested U-shapes (Figure 1). Equestrian jump stands, wooden rails, and barrier tape were used as barriers for the labyrinth (Figure 2). The starting point was marked with two cavaletti jumps, which were positioned in an intermittent V-shape (Figure 1). A bucket with a reward (a handful of concentrate) was placed in the middle of the labyrinth (Figure 1 and Figure 2).

### 2.4. Training Phase

Horses were habituated to the barriers of the labyrinth by leading them each through an L-shaped labyrinth 10 times on 5 days; no food reward was present during the habituation. The operant conditioning to the neon-green bucket (Ø 28 cm) was carried out during a period of 4 weeks by feeding each horse a handful of concentrate from the bucket once a day in their individual box stalls.

### 2.5. Experimental Procedure

Horses were tested individually in the same order every day. Experiments always took place between 2 h after the last and 1 h before the next feeding time. During testing, subjects were visually isolated from other conspecifics but remained in auditory and olfactory contact. Each horse was tested on two consecutive test days. Each test day consisted of 5 consecutive trials, resulting in 10 trials per horse. For each trial, the horse was led with a lead rope to the starting point by the experimenter and an assistant (re)filled the food reward in the bucket visibly to the horse and then positioned himself at the wall sideways from the starting point. After waiting for another 5 s, the horse was released by removing the lead rope from the headcollar. 

The 16 horses were randomly allocated to two test groups of 8 horses each, which experienced one of two different treatments:

- No demonstrator: After releasing the horse at the starting point, the experimenter stepped back sideways behind the cavaletti jumps.
- Human demonstrator: After releasing the horse at the starting point, the experimenter immediately started walking towards the rewarded bucket without further interacting with the horse. As soon as the human demonstrator started moving, the horse was free to solve the detour in its own pace and to choose its own direction, i.e., left or right side of the detour task. The human demonstrator always chose the direction to the right of the barriers and reached the rewarded bucket within an approximate latency of 30 s.

### 2.6. Data Recording and Analysis

All data were recorded directly by one observer; all trials were video recorded for controls. Latency between the release of the horse at the starting point and the horse touching the rewarded bucket served as outcome variable. If a horse did not obtain the food reward within 180 s, i.e., was unsuccessful to detour the obstacle within 180 s, the trial was terminated by leading the horse back to the starting point. In this case, a latency of 180 s was recorded and the trial was classified as unsuccessful.

Statistical analysis was conducted in R (version 3.4.3; [29]). The outcome variables ‘occurrence of unsuccessful trial’ (dichotomised: yes, no) and ‘latency’ were analysed by using generalised linear mixed-effects models (glmer) and linear mixed-effects models (lmer), respectively (package lme4; [30]). The explanatory variables included treatment (factor with 2 levels: no demonstrator, human demonstrator), test day (factor with 2 levels: 1, 2), trial (factor with 5 levels: 1, 2, 3, 4, 5), and their interactions (3-way and all possible 2-way) as fixed effects and, to account for dependencies in the data structure, test day nested in the horse nested in the test group as random effect. Model assumptions were checked by graphical analysis of residuals (normal distribution, homoscedasticity); the outcome variable ‘latency’ was log transformed. The final model was accomplished by a stepwise backwards reduction (the smallest model included the main effects only) with a *p*-value of 0.05 as criterion of exclusion and model estimates and 95% confidence intervals of the fixed effects were calculated.

## 3. Results

In the test group without demonstration, 14 trials by 4 horses (test day 1: 10 trials by 3 horses; test day 2: 4 trials by 3 horses) were unsuccessful, whereas only 1 horse was unsuccessful once (in the first trial on test day 1) in the test group with a human demonstrator. However, the occurrence of unsuccessful trials was not affected by the treatment (*p* = 0.13), test day (*p* = 0.29), or trial (*p* = 0.4).

**Figure 3:**
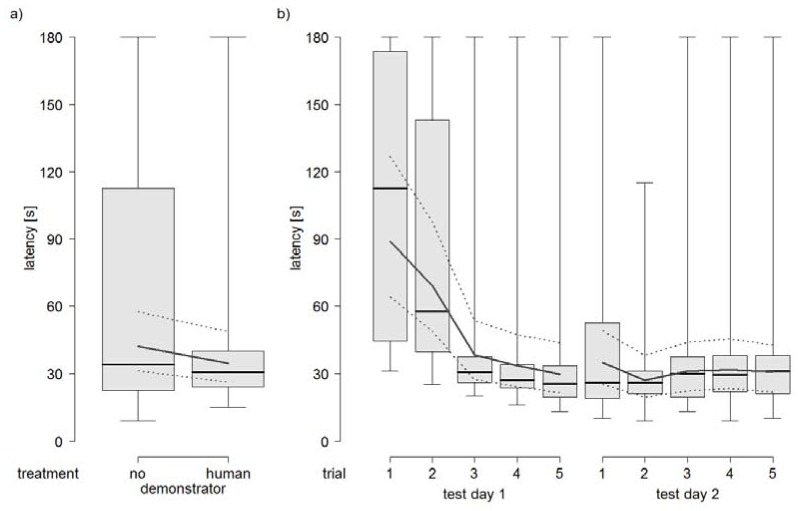
Latency to reach the rewarded bucket in (**a**) the two different treatments and (**b**) trial 1 to 5 on test day 1 and 2.

Horses did not show shorter latencies in solving the detour task with a human demonstrator in comparison with no demonstrator (treatment: *p* = 0.39; Figure 3a). However, in both test groups, the latency to reach the rewarded bucket decreased from trial 1 to 3 and levelled off from trial 3 to 5 on test day 1, whereas it remained on an equivalent level in trial 1 to 5 on test day 2 (test day × trial: *p* < 0.001; Figure 3b).

## 4. Discussion

We investigated the ability of horses to socially learn from humans in a spatial problem-solving task. Contrary to our hypothesis, we did not find that horses which observed a demonstration by a human solved a detour task faster than those without a demonstration [10]. However, horses in both test groups improved over trials, a finding which is in line with previous studies on spatial problem-solving in horses [19,25]. Our results indicate that horses do not prefer the use of social information provided by humans when being confronted with a spatial task. The use of social information in horses thus seems to be context-specific and limited to instrumental tasks [5,9,12].

Horses are very sensitive in interpreting human communicative and attention cues. They use human pointing gestures to find food [31] and adjust their begging behaviour to the attentive states of humans [32]. Horses also tend to choose a potentially baited container when it is located next to a human, independent of the person’s attentive state, indicating that horses can use humans as a local enhancement cue alone [33]. In the current study, however, observing a human demonstrating how to detour an obstacle did not affect the horses’ detour performance. This is surprising, given horses’ inclination to attend to even subtle human cues [34]. However, our findings are in agreement with the performance of other domestic ungulates in spatial problem-solving tasks using conspecific demonstrators; e.g., horses in similar detour tasks [10] or goats in maze-learning tasks [35]. Human demonstration, in turn, led to improved detour performances in goats and dogs [19,20], raising the question why horses did not improve with demonstration in a similar task.

A possible explanation for our inability to find an effect of human demonstration on the detour performance in horses might be a potential floor effect of the latency over trials for both test groups. Dogs and goats only showed moderate or no improvement over trials in similar spatial tasks [19,20]. Horses from both test groups, however, rapidly improved in detouring the obstacle with latencies levelling off after the third trial of the first test day. Rapid individual improvement in detour tasks has been previously shown for horses, indicating that only a few trials are necessary for horses to learn a new spatial task [23,25]. Thus, our negative findings for both treatment groups might appear because the horses were simply not able to solve the detour any faster, which masked a potential treatment effect between test groups. This rapid improvement needs to be taken into account for handling practices, but also for designing future experimental studies. Adding more complexity to a spatial problem-solving task might improve the detection of potential treatment effects by conspecific or human demonstration.

## 5. Conclusions

Our results show that horses do not use information from humans in a spatial problem-solving task. The use of social information in horses thus seems to be context-specific and limited to instrumental tasks.
